# Immune checkpoint inhibitors across neoadjuvant, perioperative, and adjuvant settings for resectable non-small-cell lung cancer: a systematic review and meta-analysis of randomized trials

**DOI:** 10.3389/fphar.2026.1949219

**Published:** 2026-09-28

**Authors:** Jincan Dai, Mengni Liu, Hu Li, Yao Wang

**Affiliations:** 1 Dali Bai Autonomous Prefecture People’s Hospital, Dali, Yunnan, China; 2 The Seventh Retired Cadres Rest Center of Jinan, Shandong Provincial Military Command, Jinan, Shandong, China; 3 Jinan Fifth People’s Hospital Affiliated to Shandong Second Medical University, Jinan, Shandong, China

**Keywords:** adjuvant, immunotherapy, neoadjuvant immunotherapy, non-small cell lung cancer, overall survival, pathological response

## Abstract

**Background:**

Randomized evidence for immune checkpoint inhibition in resectable non-small-cell lung cancer has progressed from pathological response to mature survival outcomes. We examined exploratory directional alignment across endpoints and quantified clinical yield, treatment burden, and the extent of perioperative therapy delivery.

**Methods:**

We synthesized 20 peer-reviewed reports from 12 parent randomized trials, including 8,197 unique participants and 13 eligible randomized treatment comparisons. The parent trial was the analysis unit, and the latest compatible report replaced earlier estimates for each time-to-event outcome. Direct randomized comparisons were pooled using restricted maximum-likelihood random-effects models. We reconstructed cumulative event-free survival (EFS) and overall survival (OS) evidence, estimated absolute differences, performed exploratory trial-level cross-endpoint analyses, and pooled the proportion of experimental-arm patients who started postoperative study treatment.

**Results:**

Across seven trials, treatment initiated before surgery reduced EFS events by 41% (HR, 0.59; 95% CI: 0.53–0.66; I^2^ = 12%). Five trials showed a 32% reduction in death (HR, 0.68; 95% CI: 0.58–0.80; I^2^ = 0%). Per 100 randomized patients, preoperative-start therapy produced 31 additional major pathological responses (95% CI: 24–39), 22 additional pathological complete responses (95% CI: 16–27), and 4 additional patients who underwent or attempted curative-intent surgery (95% CI: 1–7). Grade ≥3 treatment-related adverse events increased by approximately 4 per 100 patients (95% CI: 0–8). In exploratory analyses, larger pCR improvements tended to accompany lower EFS HRs (Spearman ρ = −0.67; *p* = 0.098; k = 7), and EFS and OS estimates were directionally aligned (ρ = 0.95; *p* = 0.051; k = 4). In six perioperative trials, 69.7% (95% CI: 65.5%–73.7%) of randomized experimental-arm patients started postoperative study treatment.

**Conclusion:**

ICI-containing strategies initiated before surgery improve pathological response, EFS, and OS in resectable NSCLC. Approximately 70% of patients assigned to perioperative experimental arms started postoperative study treatment; this descriptive treatment-delivery finding does not establish whether postoperative ICI can be safely omitted. Prospective response- and residual-risk-guided randomized trials are required.

## Introduction

Surgery is the foundation of curative treatment for resectable non-small-cell lung cancer (NSCLC), yet recurrence remains common in node-positive and stage III disease. Immune checkpoint inhibitors (ICIs) have, therefore, moved rapidly into neoadjuvant, perioperative, and adjuvant treatment.

Early randomized reports focused on major pathological response (MPR), pathological complete response (pCR), and event-free survival (EFS). Several trials now report mature overall survival (OS) results (Forde et al., 2025; Spicer et al., 2024; Wang et al., 2026). This evolution permits assessment of whether trial-level treatment effects on early tumor clearance, recurrence prevention, and survival show directional alignment while recognizing that such ecological comparisons cannot establish surrogacy or causality.

A second question arises from the structure of perioperative regimens. Surgery, postoperative recovery, toxicity, and recurrence may prevent patients from receiving the postoperative component. The effect estimated by intention-to-treat analysis, therefore, reflects a strategy whose postoperative delivery is incomplete. Quantifying this delivery is necessary to interpret what the randomized trials have actually established.

We performed a trial-level meta-analysis that linked all reports from the same randomized trial, replaced earlier outcome versions with mature compatible estimates, and avoided treatment ranking in the absence of direct active comparisons. The objectives were to characterize EFS-to-OS evidence maturation, quantify absolute clinical yield and burden, explore cross-endpoint directional alignment, and describe the proportion of randomized patients who entered postoperative study treatment.

## Methods

### Review design, information sources, and eligibility

The review followed PRISMA 2020 principles (Page et al., 2021). Study identification was reconstructed from an archived, already deduplicated corpus of 3,339 records with evidence available through July 11, 2026, comprising 3,160 PubMed/MEDLINE records and 179 records unique to Europe PMC after cross-source deduplication. Targeted update searches of PubMed/MEDLINE, Europe PMC, ClinicalTrials.gov, reference lists, and forward citations were conducted through August 16, 2026, and one eligible report was added. No language restriction was applied to the update search. Because the historical source-level exports, exact database-specific Boolean strings, and duplicate-removal log were not retained, this process was a forensic reconstruction rather than a *de novo* rerun of every original database search. Reconstructed Embase and CENTRAL strategies are provided in the Supplementary Methods but were not rerun because subscription access was unavailable.

Eligible studies randomly assigned adults with resectable NSCLC to an ICI-containing neoadjuvant, perioperative, or adjuvant strategy versus a non-ICI control. Conference abstracts and trial registries were used for surveillance, whereas quantitative synthesis required a peer-reviewed full report. Nonrandomized cohorts, single-arm studies, biomarker-only reports, and reports fully superseded for the same endpoint without an independent outcome contribution were excluded from primary pooling.

One reviewer rescreened all 3,340 records and documented screening counts and report-level eligibility decisions. Sixty reports underwent report-level eligibility assessment; 40 were excluded with prespecified reasons, leaving 20 peer-reviewed reports from 12 parent randomized trials and 13 eligible randomized treatment comparisons (Figure 1). Full search strings, screening codes, and report-level exclusion reasons are documented in the Supplementary Methods and the Search and Screening Audit Workbook.

**FIGURE 1:**
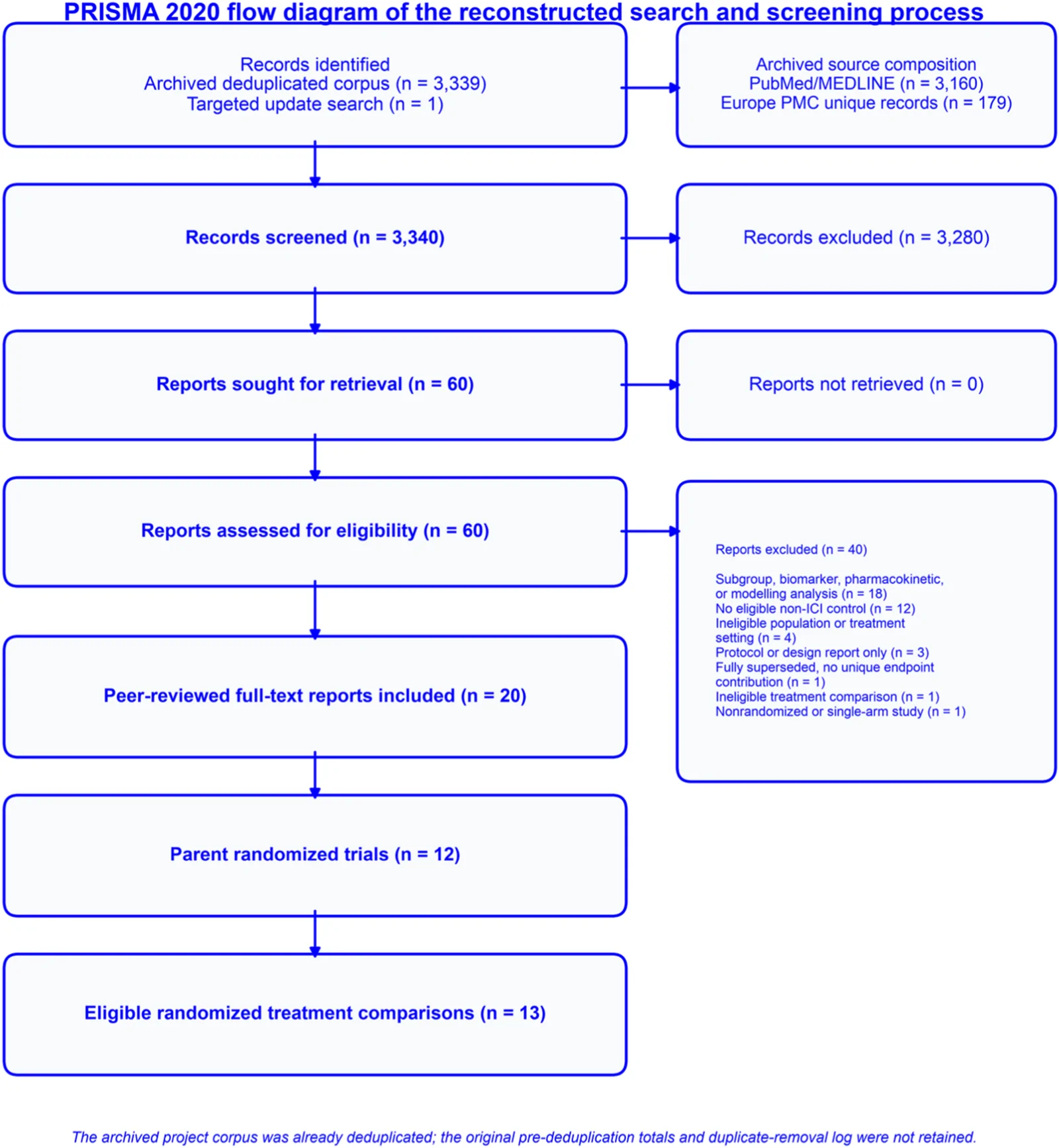
PRISMA 2020 flow diagram of the reconstructed search and screening process. The archived project corpus was already deduplicated; original pre-deduplication totals and the historical duplicate-removal log were unavailable.

### Trial and report version framework

The parent randomized trial was the analysis unit. Reports were linked by trial identifier, population, treatment arms, endpoint definition, and data cutoff. For each time-to-event outcome, a mature, compatible report replaced the earlier estimate rather than contributing a second weight. Earlier primary reports and dedicated surgical reports remained eligible for pathological response, surgery, safety, and treatment-delivery data not replaced in later publications (Felip et al., 2021; Mitsudomi et al., 2025; Liberman et al., 2026; Awad et al., 2025; Fang et al., 2026). The endpoint-level trial–report map, including the selected report, data cutoff, and endpoint definition for each trial and outcome, is provided in Supplementary Table S1.

To describe evidence maturation, we ordered primary and updated reports by publication date. At each step, the pooled estimate contained the most recent compatible result available for every trial. This produced a cumulative sequence without double-counting report versions.

### Outcomes and clinical estimands

The main survival estimands were EFS and OS for strategies initiated before surgery. We defined “preoperative-start” as an overarching timing category because neoadjuvant-only and perioperative regimens both expose the intact tumor to ICI before resection. This pooled category evaluates the common feature of preoperative initiation; it does not imply clinical equivalence between the regimens or isolate the effect of postoperative ICI continuation. Timing-specific neoadjuvant-only, perioperative, and adjuvant-only estimates were, therefore, retained as complementary analyses. NADIM II progression-free survival was included only in an endpoint-compatible sensitivity analysis.

Binary efficacy outcomes were MPR, pCR, and whether a patient underwent or attempted curative-intent surgery. This surgical estimand was distinguished from completed tumor resection and R0 resection, which were retained descriptively when definitions were incompatible across trials. Randomized or prespecified modified intention-to-treat denominators were used; patients without surgery were considered nonresponders when this matched the trial analysis. Safety outcomes were grade ≥3 treatment-related adverse events (TRAEs) and adverse-event-related treatment discontinuation; eligibility for each safety synthesis required compatible attribution, denominator, and reporting-window definitions. Trial-level safety definitions, populations, reporting windows, denominators, and inclusion decisions are provided in Supplementary Table S4.

Postoperative delivery was defined as the proportion of all randomized experimental-arm patients who started postoperative study treatment. This denominator preserves the treatment strategy initiated at randomization. Initiation was distinguished from completion when reported. Because initiation is a post-randomization treatment-delivery variable, the pooled proportion describes implementation of the assigned perioperative strategy and does not estimate the causal effect of postoperative continuation versus omission.

### Statistical analysis

Hazard ratios (HRs) and risk ratios were analyzed on the logarithmic scale. Standard errors were calculated according to the confidence level reported by each trial. Inverse-variance random-effects models used restricted maximum likelihood. Heterogeneity was described using tau^2^, Cochran Q, and I^2^. Modified Hartung–Knapp intervals were retained as sensitivity analyses (Röver et al., 2015). Fixed-effect estimates, modified Hartung–Knapp intervals, leave-one-out analyses, and prediction intervals, where estimable, are reported in Supplementary Table S3.

Absolute risk differences (RDs) were pooled for MPR, pCR, surgery, grade ≥3 TRAEs, and discontinuation. Numbers needed to treat (NNT) or harm (NNH) were calculated as the inverse of the pooled absolute difference and interpreted as approximate when the RD confidence interval approached the null. Postoperative initiation proportions were pooled on the logit scale.

Cross-endpoint directional alignment was explored using Spearman correlation of trial-level log HRs for EFS and OS and absolute treatment differences for pathological response. These ecological analyses were hypothesis-generating, were not designed to validate a surrogate endpoint, and cannot establish a causal pathway between pathological response and survival. No network ranking was performed because the active-treatment network contained no closed loop.

### Risk of bias

Risk of bias was evaluated using the Cochrane RoB 2 framework (Sterne et al., 2019), considering randomization, deviations from assigned treatment, missing data, outcome measurement, and selective reporting. Domain-level judgments and supporting rationales are provided in Supplementary Table S2.

## Results

### Study characteristics

The 20 peer-reviewed reports represented 12 parent randomized trials, 8,197 unique randomized participants, and 13 eligible randomized treatment comparisons (Figure 1; Table 1). The update added a dedicated NeoTORCH surgical-outcomes report and identified the concurrently randomized CheckMate 816 nivolumab-plus-ipilimumab comparison (Awad et al., 2025; Fang et al., 2026). Because 108 chemotherapy controls overlapped with the main CheckMate 816 comparison, the dual-ICI comparison was summarized qualitatively and was not included as an independent weight in the existing pooled models. Two parent trials evaluated neoadjuvant-only therapy, six evaluated perioperative therapy, and four evaluated adjuvant-only ICI. Ten were phase III trials, and two were randomized phase II trials.

**TABLE 1:** Characteristics of the included randomized trials.

Trial	Timing	N	Stage/population	ICI strategy	Primary contribution
CheckMate 816	Neoadjuvant	471*	IB–IIIA	Nivolumab + CT; nivolumab + ipilimumab	EFS, OS, MPR, pCR, surgery
TD-FOREKNOW	Neoadjuvant	94	IIIA–IIIB (T3N2)	Camrelizumab + CT	EFS, MPR, pCR, surgery
NADIM II	Perioperative	86	IIIA–IIIB	Nivolumab + CT; adjuvant nivolumab after R0	PFS, OS, MPR, pCR, surgery
KEYNOTE-671	Perioperative	797	II–IIIB (N2)	Pembrolizumab + CT; adjuvant pembrolizumab	EFS, OS, MPR, pCR, surgery
AEGEAN	Perioperative	802	II–IIIB (N2)	Durvalumab + CT; adjuvant durvalumab	EFS, MPR, pCR, surgery
NeoTORCH	Perioperative	501	II–III; primary report stage III	Toripalimab + CT; postoperative continuation	EFS, OS, MPR, pCR, surgery
CheckMate 77T	Perioperative	461	IIA–IIIB	Nivolumab + CT; adjuvant nivolumab	EFS, MPR, pCR, surgery
RATIONALE-315	Perioperative	453	II–IIIA	Tislelizumab + CT; adjuvant tislelizumab	EFS, OS, MPR, pCR, surgery
IMpower010	Adjuvant	1,005	IB–IIIA after CT	Atezolizumab	DFS, OS
PEARLS/KEYNOTE-091	Adjuvant	1,177	IB–IIIA after resection ± CT	Pembrolizumab	DFS
BR.31	Adjuvant	1,415	IB–IIIA after resection ± CT	Durvalumab	DFS
ANVIL	Adjuvant	935	IB–IIIA after planned therapy	Nivolumab	DFS

* CheckMate 816 included two eligible randomized comparisons. The 221-patient concurrently randomized nivolumab-plus-ipilimumab comparison included 108 chemotherapy controls who were also represented in the main comparison; therefore, 471 unique participants were counted. The dual-ICI comparison was summarized qualitatively and was not entered as an independent weight in the existing pooled models (Awad et al., 2025).

CT, platinum-based chemotherapy; DFS, disease-free survival; EFS, event-free survival; ICI, immune checkpoint inhibitor; MPR, major pathological response; OS, overall survival; pCR, pathological complete response.

Eight trials had low risk of bias for the primary efficacy outcome. Four had some concerns, mainly because time-to-event outcomes were investigator-assessed in open-label trials. No trial was judged to be at high risk. Trial-level domain judgments are shown in Supplementary Table S2 (Forde et al., 2022; Lei et al., 2023; Provencio et al., 2023; Wakelee et al., 2023; Heymach et al., 2023; Lu et al., 2024; Cascone et al., 2024; Yue et al., 2025; Felip et al., 2025; O'Brien et al., 2022; Goss et al., 2026; Chaft et al., 2026).

### Evidence maturation from EFS to OS

Seven trials contributed EFS data for a strategy initiated before surgery. The pooled HR was 0.59 (95% CI: 0.53–0.66; *p* < 0.001), with low heterogeneity (I^2^ = 12%). The estimate remained within a narrow beneficial range as new trials and mature reports entered the evidence set (Figure 2). Fixed-effect and modified Hartung–Knapp results were directionally consistent, and the benefit was retained in every leave-one-out analysis (Supplementary Table S3). The principal pooled efficacy, clinical-yield, treatment-burden, and treatment-delivery estimates are summarized in Table 2, and mature trial-specific OS landmarks are presented in Table 3.

**FIGURE 2:**
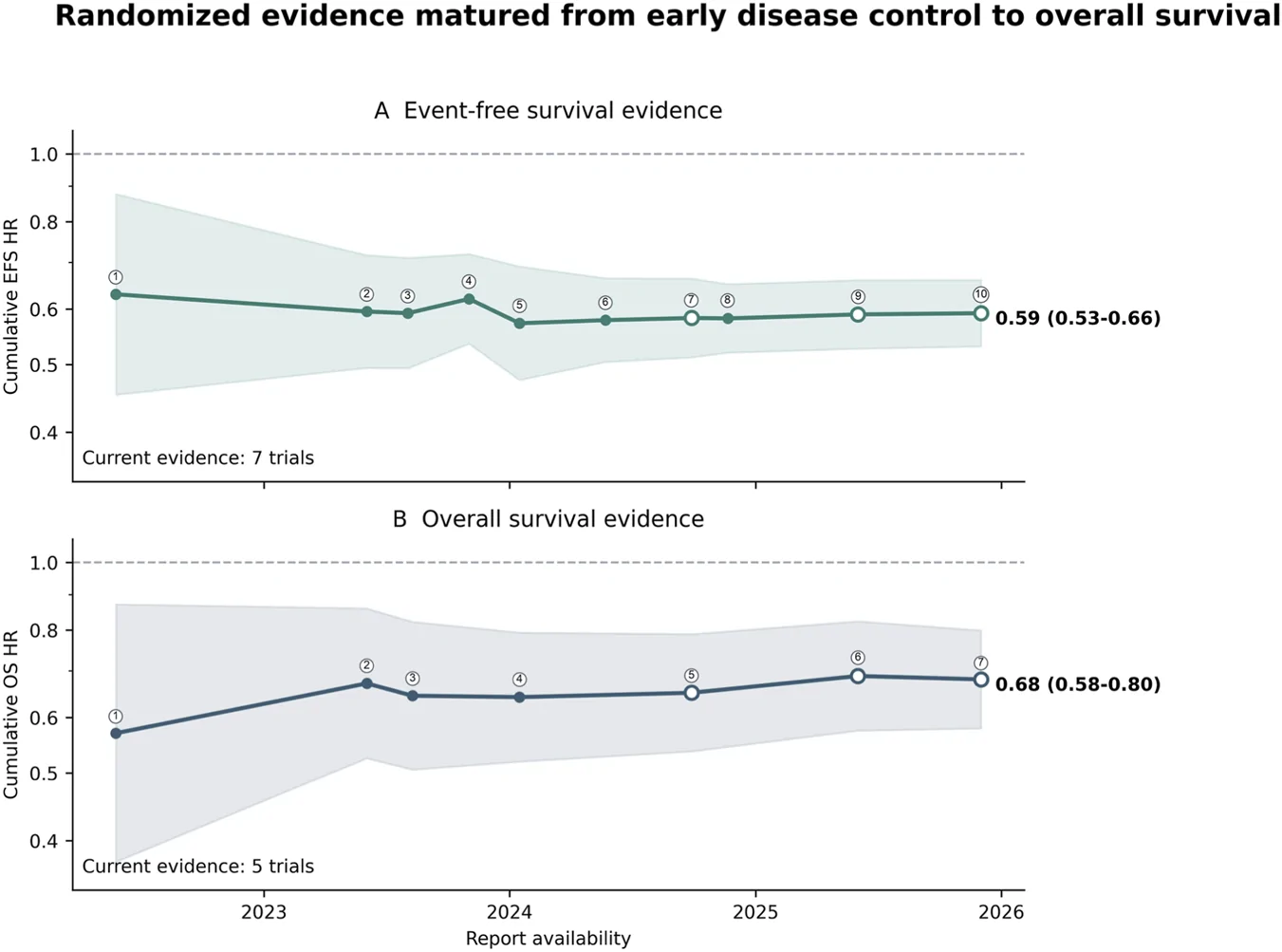
**(A)** Maturation of pooled EFS evidence and **(B)** maturation of pooled OS evidence as new trials and updated reports became available. Numbered points identify the trial/report added or updated. **(A)** EFS: 1, CheckMate 816 (Forde, 2022); 2, KEYNOTE-671 (Wakelee, 2023); 3, TD-FOREKNOW (Lei, 2023); 4, AEGEAN (Heymach, 2023); 5, NeoTORCH (Lu, 2024); 6, CheckMate 77T (Cascone, 2024); 7, KEYNOTE-671 update (Spicer, 2024); 8, RATIONALE-315 (Yue, 2025); 9, CheckMate 816 update (Forde, 2025); 10, RATIONALE-315 update (Wang, 2026). **(B)** OS: 1, CheckMate 816 interim (Forde, 2022); 2, NADIM II (Provencio, 2023); 3, KEYNOTE-671 (Spicer, 2024); 4, NeoTORCH (Lu, 2024); 5, RATIONALE-315 (Yue, 2025); 6, CheckMate 816 final (Forde, 2025); 7, RATIONALE-315 final (Wang, 2026). At each date, the latest compatible estimate replaced the earlier report from the same trial.

**TABLE 2:** Main evidence-maturation and clinical-delivery results.

Domain	Outcome	k	Pooled estimate (95% CI)	Clinical expression
Survival	Preoperative-start EFS	7	HR 0.59 (0.53–0.66)	41% lower event hazard
Survival	Preoperative-start OS	5	HR 0.68 (0.58–0.80)	32% lower death hazard
Clinical yield	MPR	8	RD +31.2% (+23.7 to +38.7)	NNT 3.2
Clinical yield	pCR	8	RD +21.8% (+16.3 to +27.3)	NNT 4.6
Clinical yield	Underwent or attempted curative-intent surgery	8	RD +4.3% (+1.2 to +7.4)	More patients underwent or attempted surgery
Treatment burden	Grade ≥3 TRAE	6	RD +4.2% (+0.1 to +8.4)	NNH 23.5
Treatment burden	AE-related discontinuation	6	RD +5.4% (+2.5 to +8.2)	NNH 18.6
Delivery	Started postoperative treatment	6	69.7% (65.5–73.7)	Of randomized experimental arm
Adjuvant-only	DFS	4	HR 0.86 (0.78–0.95)	14% lower recurrence/death hazard

AE, adverse event; DFS, disease-free survival; EFS, event-free survival; HR, hazard ratio; MPR, major pathological response; NNH, number needed to harm; NNT, number needed to treat; OS, overall survival; pCR, pathological complete response; RD, risk difference; TRAE, treatment-related adverse event.

**TABLE 3:** Mature overall-survival landmarks in preoperative-start trials.

Trial	Strategy	Landmark	Experimental	Control	Absolute difference
CheckMate 816	Neoadjuvant	5-year OS	65.4%	55.0%	+10.4 percentage points
KEYNOTE-671	Perioperative	3-year OS	71%	64%	+7 percentage points
NADIM II	Perioperative	2-year OS	85.0%	63.6%	+21.4 percentage points
RATIONALE-315	Perioperative	3-year OS	79.3%	69.3%	+10.0 percentage points

Five trials contributed an OS HR. The pooled HR was 0.68 (95% CI: 0.58–0.80; *p* < 0.001), with no detected heterogeneity (I^2^ = 0%). The OS evidence evolved from the initial CheckMate 816 signal to concordant results from KEYNOTE-671, NADIM II, NeoTORCH, and final RATIONALE-315. Mature landmarks showed absolute OS differences of 10.4 percentage points at 5 years in CheckMate 816, 7 points at 3 years in KEYNOTE-671, and 10 points at 3 years in RATIONALE-315.

### Absolute clinical yield and treatment burden

Across eight preoperative-start trials, ICI produced an absolute MPR increase of 31.2 percentage points (95% CI: 23.7–38.7; NNT 3.2) and a pCR increase of 21.8 points (95% CI: 16.3–27.3; NNT 4.6). The probability of undergoing or attempting curative-intent surgery increased by 4.3 points (95% CI: 1.2–7.4).

Across six trials with harmonizable definitions, grade ≥3 TRAEs increased by 4.2 points (95% CI: 0.1–8.4; approximate NNH 23.5). The broad adverse-event discontinuation estimate increased by 5.4 points (95% CI: 2.5–8.2; NNH 18.6). Thus, per 100 randomized patients, treatment yielded approximately 31 additional MPRs, 22 additional pCRs, and 4 additional patients who underwent or attempted curative-intent surgery, alongside an estimated 4 additional grade ≥3 TRAEs and 5 additional discontinuations. Safety estimates remain subject to differences in trial attribution rules, denominators, and reporting windows (Figure 3). Endpoint-specific sensitivity results and the strict-attribution safety analysis are provided in Supplementary Tables S3 and S4.

**FIGURE 3:**
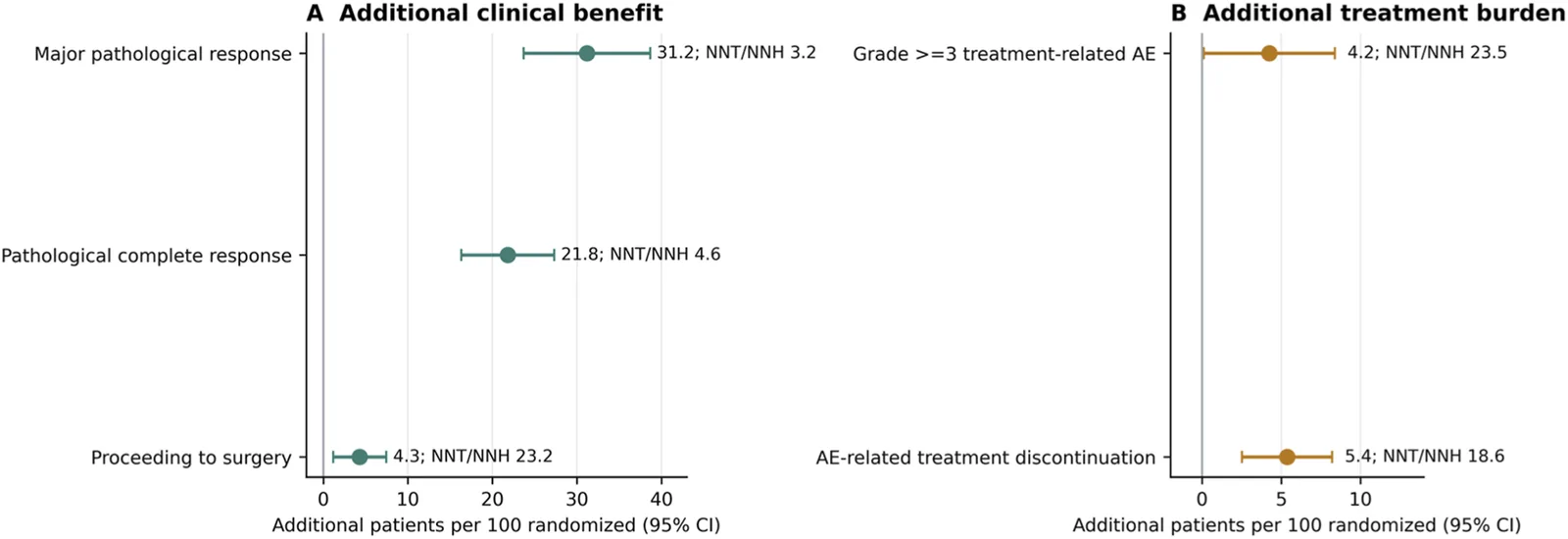
**(A)** Absolute clinical yield and **(B)** treatment burden per 100 randomized patients. Points show pooled risk differences, and horizontal lines show 95% confidence intervals.

### Cross-endpoint concordance

In exploratory trial-level analyses, larger pCR improvements tended to be associated with lower EFS HRs, although the association did not reach conventional statistical significance (Spearman ρ = −0.67; *p* = 0.098; k = 7; Figure 4). EFS and OS estimates were directionally aligned across four trials with both outcomes available (ρ = 0.95; *p* = 0.051; k = 4), but the small number of trials precluded firm inference.

**FIGURE 4:**
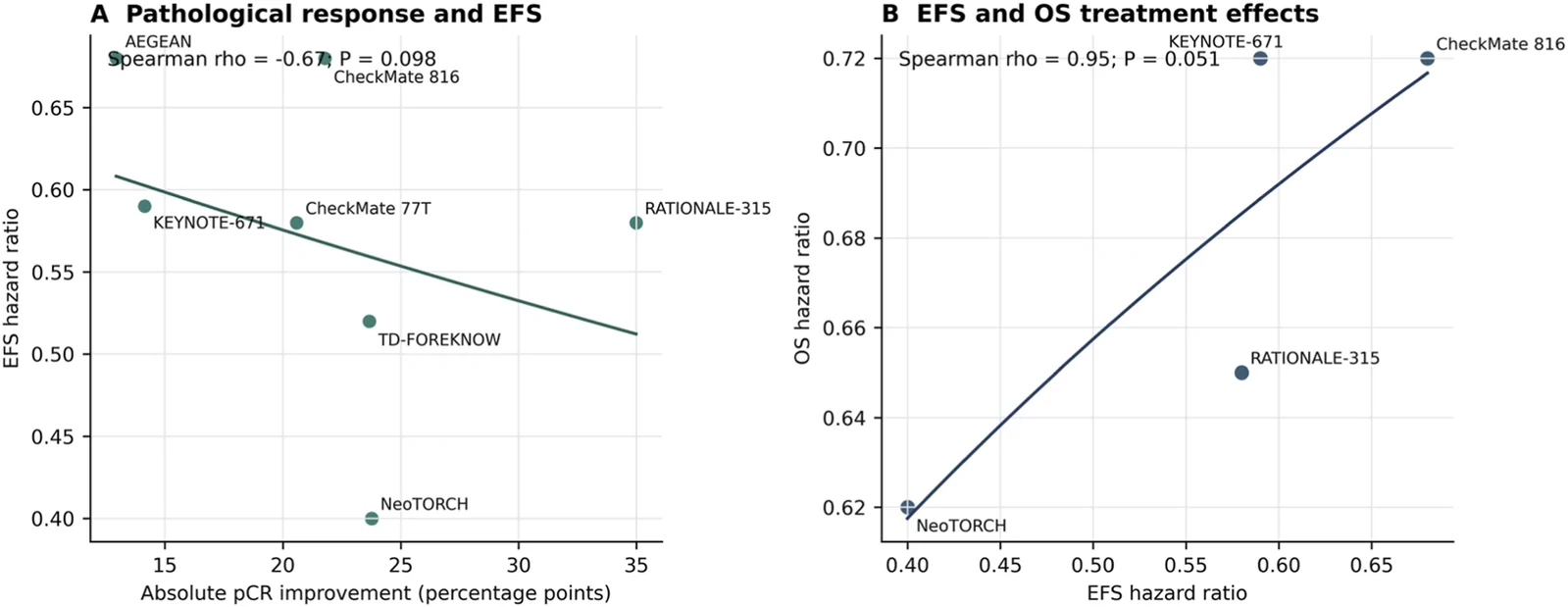
Exploratory trial-level directional alignment between **(A)** pCR and EFS treatment effects (k = 7; Spearman ρ = 0.67; p = 0.098) and **(B)** EFS and OS treatment effects (k = 4; ρ = 0.95; p = 0.051). These ecological analyses were not designed for surrogate validation or causal inference.

### Realized postoperative treatment delivery

Six perioperative trials reported sufficient information on postoperative initiation. The pooled proportion of randomized experimental-arm patients who started postoperative study treatment was 69.7% (95% CI: 65.5%–73.7%), ranging from 62.0% in CheckMate 77T to 74.3% in RATIONALE-315 (Figure 5). In every trial, the proportion entering postoperative treatment was lower than the proportion proceeding to surgery. This descriptive attrition occurred after randomization; it neither estimates the independent contribution of postoperative ICI nor identifies patients in whom continuation can be safely omitted.

**FIGURE 5:**
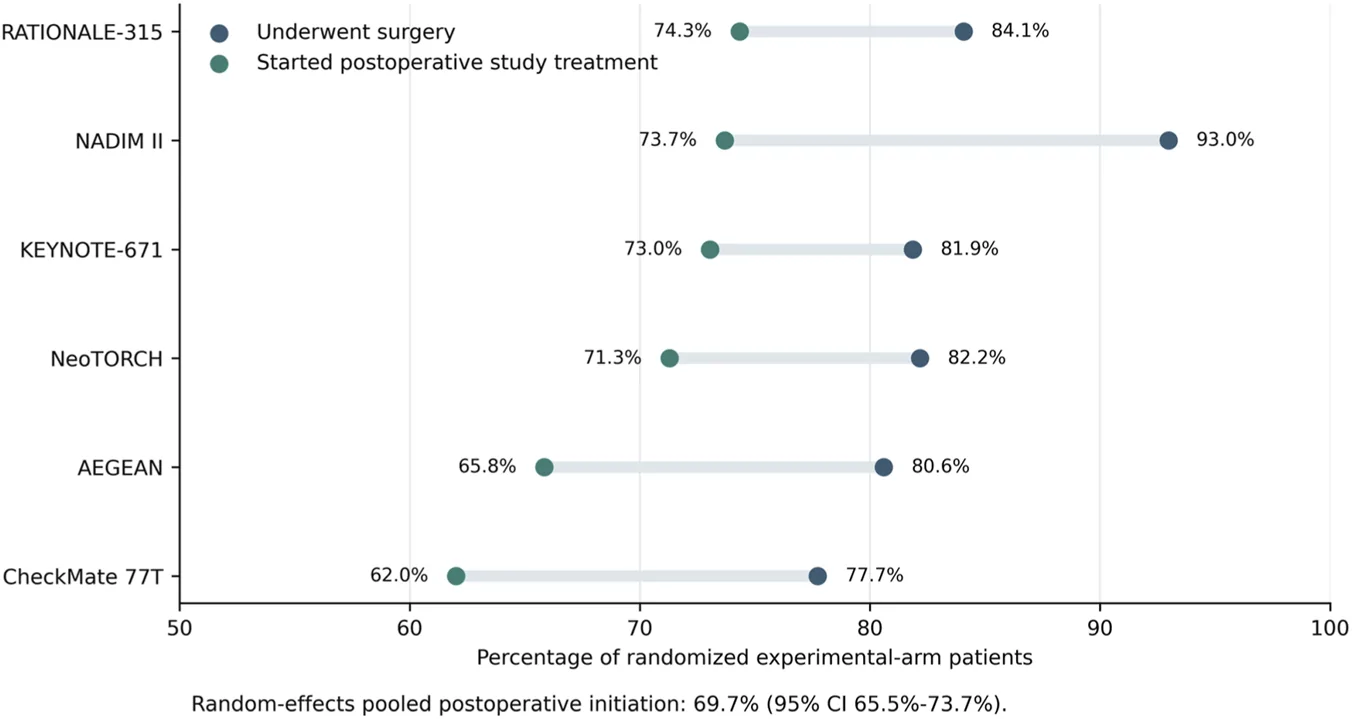
Surgical delivery and initiation of postoperative study treatment among patients randomized to the experimental perioperative strategy. Initiation is a descriptive post-randomization variable and does not estimate the causal effect of postoperative continuation versus omission.

### Timing-specific complementary analyses

Perioperative trials showed EFS HR 0.58 (95% CI: 0.51–0.66) and OS HR 0.67 (95% CI: 0.55–0.80). Neoadjuvant-only trials showed EFS HR 0.66 (95% CI: 0.50–0.87), and mature CheckMate 816 data confirmed an OS benefit (HR, 0.72; 95% CI: 0.523–0.998). Across four adjuvant-only trials, the pooled DFS HR was 0.86 (95% CI: 0.78–0.95). Comparisons across treatment settings are indirect because trial populations, control regimens, endpoint definitions, and follow-up differ.

## Discussion

This meta-analysis shows that the randomized evidence has crossed an important threshold. The early increase in pathological response is now accompanied by a sustained reduction in recurrence and a reproducible OS benefit. When ICI was initiated before surgery, EFS events were reduced by 41% and deaths by 32%, with minimal between-trial heterogeneity. The result is consistent across different antibodies, chemotherapy backbones, and trial populations.

The absolute-effect analysis makes the magnitude clinically tangible. For every 100 randomized patients, preoperative-start therapy produced about 31 additional MPRs and 22 additional pCRs while preserving or slightly increasing the likelihood of surgery. The corresponding excess was about four severe TRAEs. This balance supports preoperative ICI as an efficient curative-intent strategy rather than an intervention whose benefit depends only on a relative scale.

The trial-level cross-endpoint analyses were exploratory. Larger pathological-response improvements tended to accompany greater EFS effects, and EFS and OS estimates were directionally aligned. However, the small number of trials, ecological nature of the analysis, differences in follow-up, and absence of individual-patient data preclude surrogate validation or causal inference. Treatment delivered while the primary tumor and regional lymph nodes remain present may plausibly support systemic immune priming, but this biological rationale does not establish a causal pathway from pathological response to survival.

The pooled postoperative initiation proportion describes implementation of the assigned perioperative strategy rather than the efficacy of its postoperative component. Patients who did not start postoperative treatment were selected after randomization through events such as failure to undergo surgery, postoperative recovery, toxicity, progression, and clinical decision-making. Consequently, comparisons between patients who did and did not receive postoperative treatment would be subject to substantial post-randomization selection bias. The present analysis supports prospective randomized evaluation of postoperative de-escalation but does not support omission of postoperative ICI in routine practice.

Adjuvant-only ICI reduced DFS events in pooled randomized evidence, but its numerical effect should not be directly compared with preoperative-start estimates because the comparisons are indirect. Starting treatment before surgery offers an earlier opportunity to control micrometastatic disease and provides a pathological readout, whereas postoperative-only therapy remains relevant in appropriately selected patients. The pooled overall-population estimates should not be directly extrapolated to patients with sensitizing EGFR alterations, ALK rearrangements, or other oncogene-driven tumors because molecular eligibility and subgroup representation varied across trials, and expected ICI benefit may differ.

The analysis used the parent trial rather than the report as its unit, followed outcome versions through mature updates, and retained randomized denominators for pathological and surgical endpoints. Limitations include the small number of trials in exploratory correlations, residual differences in safety definitions, indirect comparisons across treatment settings, absence of individual-patient data, and incomplete reporting of postoperative treatment completion and reasons for non-initiation. In addition, historical source-level exports and the original deduplication log were unavailable, Embase and CENTRAL were not rerun, and the reconstructed screening was performed by a single reviewer. Independent verification and subscription-database reruns remain necessary before final resubmission.

## Conclusion

Immune checkpoint inhibitor strategies initiated before surgery improve pathological response and reduce recurrence and mortality in resectable NSCLC. Adjuvant-only ICI also provides DFS benefit in pooled randomized evidence, although cross-setting comparisons remain indirect. In perioperative trials, postoperative study treatment was initiated by approximately 70% of patients assigned to the experimental strategy. This observation describes treatment attrition but does not identify the independent contribution of postoperative ICI or the patients in whom it can be safely omitted. Direct randomized trials incorporating pathological response and residual-risk biomarkers are needed to define postoperative treatment duration.

## Data Availability

The original contributions presented in the study are included in the article/Supplementary Material; further inquiries can be directed to the corresponding author.
