# Supplementary material for: Immune checkpoint inhibitors across neoadjuvant, perioperative, and adjuvant settings for resectable non-small-cell lung cancer: a systematic review and meta-analysis of randomized trials
Source: Front Pharmacol. 2026 Sep 28;17:1949219. doi: 10.3389/fphar.2026.1949219 (PMC13654980; doi:10.3389/fphar.2026.1949219)
Supplement: Supplementary file 1 [file Supplementaryfile4.docx]

**Supplementary Methods**

**Immune checkpoint inhibitors across neoadjuvant, perioperative, and adjuvant settings for resectable non–small-cell lung cancer**

Search update cutoff: August 16, 2026

Search reconstruction note: Historical source-level exports, verbatim original database strings, and the duplicate-pair log were not retained. The strategies below are reproducible reconstructions. The 3,280 title/abstract exclusions are supported at aggregate-count level, and the 60 full-text/report decisions are documented in the Search and screening audit workbook.

# 1. Review design and reporting framework

This systematic review and trial-level meta-analysis was conducted in accordance with PRISMA 2020 principles. The parent randomized trial, rather than an individual publication, was the analysis unit. Multiple publications were linked to their parent trial so that updated estimates replaced overlapping earlier versions without adding a second statistical weight.

The review addressed randomized evidence for immune checkpoint inhibitor (ICI) strategies initiated before surgery (neoadjuvant-only or perioperative) and after complete resection (adjuvant-only) in adults with resectable or completely resected non–small-cell lung cancer (NSCLC).

# 2. Eligibility criteria

| **Domain** | **Operational criterion** |
| --- | --- |
| Population | Adults with histologically confirmed resectable NSCLC before surgery or completely resected NSCLC after surgery. Trial-specific TNM editions and stage ranges were accepted and retained during extraction. |
| Intervention | A randomized strategy containing an anti–PD-1, anti–PD-L1, or other eligible ICI administered as neoadjuvant-only, perioperative, or adjuvant-only treatment, alone or with protocol-specified systemic therapy. |
| Comparator | A concurrent, randomized, non-ICI control consistent with the trial setting, including chemotherapy with or without placebo, placebo, observation, or best supportive care. Eligible additional randomized comparisons sharing a control arm were catalogued but were not treated as independent studies without shared-control adjustment. |
| Study design | Randomized phase II or III trial. Peer-reviewed full reports were required for quantitative synthesis. Registries and conference records were used for surveillance and report linkage. |
| Outcomes | EFS, DFS, PFS, OS, MPR, pCR, receipt/attempt of surgery, R0 resection where compatible, grade ≥3 treatment-related adverse events, adverse-event-related discontinuation, and initiation of the protocol-specified postoperative component. |
| Exclusions | Nonrandomized or single-arm studies; ineligible populations/settings; no eligible standard non-ICI comparator; protocols/design records without results; biomarker-only, subgroup-only, pharmacokinetic, pooled-safety, cost-effectiveness, or modelling reports without an independent prespecified trial-level endpoint; and reports fully superseded for the same endpoint without a unique contribution. |

# 3. Information sources and search dates

The retained project archive contained 3,339 already deduplicated records with evidence through July 11, 2026: 3,160 records attributed to PubMed/MEDLINE and 179 records reported as unique to Europe PMC after cross-source deduplication. The source-level exports and historical duplicate-removal log were unavailable at reconstruction.

A targeted update through August 16, 2026 used PubMed/MEDLINE, Europe PMC, ClinicalTrials.gov, backward reference checking, and forward citation tracking based on parent-trial names, registry numbers, drug names, and report DOIs. No language restriction was applied. The update added one eligible peer-reviewed report, the dedicated NeoTORCH surgical-outcomes publication, without adding a new parent trial. Embase and CENTRAL strategies were reconstructed for transparency but were not rerun because subscription access was unavailable.

# 4. Complete reconstructed search strategies

These strings document the reproducible strategy used for reconstruction and update. They must not be described as verbatim copies of the unavailable historical database queries.

## 4.1 PubMed/MEDLINE

(("Carcinoma, Non-Small-Cell Lung"[Mesh] OR NSCLC[tiab] OR "non-small cell lung"[tiab] OR "non-small-cell lung"[tiab]) AND ("Immune Checkpoint Inhibitors"[Mesh] OR immunotherap*[tiab] OR checkpoint inhibitor*[tiab] OR PD-1[tiab] OR PD-L1[tiab] OR pembrolizumab[tiab] OR nivolumab[tiab] OR atezolizumab[tiab] OR durvalumab[tiab] OR camrelizumab[tiab] OR toripalimab[tiab] OR tislelizumab[tiab] OR sintilimab[tiab]) AND (resectable[tiab] OR resected[tiab] OR operable[tiab] OR neoadjuvant[tiab] OR perioperative[tiab] OR adjuvant[tiab]) AND (randomized controlled trial[pt] OR controlled clinical trial[pt] OR random*[tiab] OR trial[tiab]))

## 4.2 Europe PMC

((TITLE_ABS:"non-small cell lung" OR TITLE_ABS:NSCLC) AND (TITLE_ABS:resectable OR TITLE_ABS:resected OR TITLE_ABS:operable OR TITLE_ABS:neoadjuvant OR TITLE_ABS:perioperative OR TITLE_ABS:adjuvant) AND (TITLE_ABS:immunotherap* OR TITLE_ABS:"checkpoint inhibitor" OR TITLE_ABS:PD-1 OR TITLE_ABS:PD-L1 OR TITLE_ABS:pembrolizumab OR TITLE_ABS:nivolumab OR TITLE_ABS:atezolizumab OR TITLE_ABS:durvalumab OR TITLE_ABS:camrelizumab OR TITLE_ABS:toripalimab OR TITLE_ABS:tislelizumab OR TITLE_ABS:sintilimab) AND (TITLE_ABS:random* OR TITLE_ABS:trial))

## 4.3 Embase (Elsevier; reconstructed, not rerun)

('non small cell lung cancer'/exp OR nsclc:ti,ab OR 'non-small cell lung':ti,ab) AND ('immune checkpoint inhibitor'/exp OR immunotherap*:ti,ab OR 'pd 1':ti,ab OR 'pd l1':ti,ab OR pembrolizumab:ti,ab OR nivolumab:ti,ab OR atezolizumab:ti,ab OR durvalumab:ti,ab OR camrelizumab:ti,ab OR toripalimab:ti,ab OR tislelizumab:ti,ab OR sintilimab:ti,ab) AND (resectable:ti,ab OR resected:ti,ab OR operable:ti,ab OR neoadjuvant:ti,ab OR perioperative:ti,ab OR adjuvant:ti,ab) AND ('randomized controlled trial'/exp OR random*:ti,ab OR trial:ti,ab)

## 4.4 Cochrane CENTRAL (reconstructed, not rerun)

([mh "Carcinoma, Non-Small-Cell Lung"] OR NSCLC:ti,ab,kw OR "non-small cell lung":ti,ab,kw) AND ([mh "Immune Checkpoint Inhibitors"] OR immunotherap*:ti,ab,kw OR "checkpoint inhibitor":ti,ab,kw OR PD-1:ti,ab,kw OR PD-L1:ti,ab,kw OR pembrolizumab:ti,ab,kw OR nivolumab:ti,ab,kw OR atezolizumab:ti,ab,kw OR durvalumab:ti,ab,kw OR camrelizumab:ti,ab,kw OR toripalimab:ti,ab,kw OR tislelizumab:ti,ab,kw OR sintilimab:ti,ab,kw) AND (resectable:ti,ab,kw OR resected:ti,ab,kw OR operable:ti,ab,kw OR neoadjuvant:ti,ab,kw OR perioperative:ti,ab,kw OR adjuvant:ti,ab,kw)

## 4.5 ClinicalTrials.gov

Condition/disease: non-small cell lung cancer. Other terms: (resectable OR resected OR operable) AND (neoadjuvant OR perioperative OR adjuvant) AND (pembrolizumab OR nivolumab OR atezolizumab OR durvalumab OR camrelizumab OR toripalimab OR tislelizumab OR sintilimab OR immune checkpoint inhibitor). Study type: Interventional. Allocation: Randomized.

# 5. Deduplication and record identity

The historical corpus was received as already deduplicated. Because the raw exports and duplicate-pair log were not retained, the original deduplication algorithm and the attribution of individual duplicate records cannot be reproduced. The recovered aggregate count was therefore treated as the starting archive, not as a newly generated database export.

For the targeted update and report-linkage check, candidate duplicates were matched in the following order: DOI; PMID or PMCID; normalized title after case, punctuation, and whitespace standardization; clinical-trial registry number; trial acronym; and concordant first author and publication year. Distinct reports from the same trial were retained as separate reports and linked to one parent-trial identifier.

# 6. Screening and full-text eligibility assessment

One reviewer performed the forensic rescreening and reconstruction. The current archive does not support a claim of dual independent screening. Screening was hierarchical: title/abstract/metadata screening was followed by report-level assessment. All 60 reports selected for report-level assessment were retrievable. One primary decision code was assigned per report to prevent double counting of exclusion reasons.

| **Code** | **Decision category** | **Rule** |
| --- | --- | --- |
| IN01 | Included report | Eligible randomized parent trial or eligible additional randomized comparison; at least one prespecified endpoint or report-linkage role. |
| EX01 | Secondary/subgroup/biomarker/PK/model | No independent prespecified trial-level comparative endpoint for this synthesis. |
| EX02 | No eligible standard non-ICI comparator | No original randomized ICI-versus-standard non-ICI contrast. |
| EX03 | Ineligible population/setting | Population or treatment setting outside the review scope. |
| EX04 | Protocol/design record only | No eligible comparative outcome data. |
| EX05 | Fully superseded | Later peer-reviewed report completely replaced the same endpoint; no unique contribution. |
| EX06 | Ineligible treatment contrast | Randomized contrast did not meet the intervention/comparator criterion. |
| EX07 | Nonrandomized/single-arm | No randomized eligible comparator. |

# 7. PRISMA accounting

The numerical reconciliation below corresponds to main-text Figure 1.

| **Stage** | **n** |
| --- | --- |
| Archived records | 3339 |
| Update records added | 1 |
| Total records screened | 3340 |
| Title/abstract/metadata exclusions | 3280 |
| Reports assessed for eligibility | 60 |
| Reports not retrieved | 0 |
| Report-level exclusions | 40 |
| Included peer-reviewed reports | 20 |
| Parent randomized trials | 12 |
| Eligible randomized treatment comparisons | 13 |

The 40 report-level exclusions comprised 18 secondary/subgroup/biomarker/pharmacokinetic/model analyses; 12 publications without an eligible standard non-ICI comparator; four ineligible populations or settings; three protocol/design records; one fully superseded report without a unique endpoint contribution; one ineligible treatment contrast; and one nonrandomized/single-arm study. Full citations and decisions are provided in the Search and screening audit workbook.

# 8. Parent-trial linkage and report-version selection

Reports were linked using trial acronym, registry number, treatment arms, randomized population, endpoint definition, and data cutoff. For each time-to-event endpoint, the latest compatible peer-reviewed result replaced earlier overlapping estimates. Earlier reports were retained when they supplied unique pathological-response, surgery, safety, or treatment-delivery information not replaced in the later report.

The CheckMate 816 nivolumab-plus-ipilimumab versus chemotherapy report was catalogued as an eligible additional randomized comparison. Because its chemotherapy control overlapped with the parent trial’s nivolumab-plus-chemotherapy comparison, it was not added to an ordinary independent-study pool. Shared-control adjustment or qualitative/separate presentation is required.

The exact report selected for EFS/DFS/PFS, OS, MPR, pCR, surgery, safety, and postoperative treatment initiation, together with data cutoffs, endpoint definitions, populations, and selection rationale, is listed in Supplementary Table S1.

# 9. Data extraction and endpoint harmonization

For each parent trial and endpoint, the audit extracted the report identifier, publication citation, data cutoff or database lock when explicitly reported, follow-up duration, endpoint label and definition, analysis population, denominator, effect measure, and role in the synthesis. A date was recorded as ‘not reported’ when it could not be verified; publication date was not substituted for a data cutoff.

| **Endpoint** | **Harmonized extraction rule** |
| --- | --- |
| EFS | Time from randomization to progression that precluded or prevented surgery, postoperative progression or recurrence (or progression without surgery), or death, according to trial-specific review rules. |
| DFS | Time from post-resection randomization to recurrence, a new primary lung cancer where specified, or death, according to the trial protocol. |
| PFS | Time from randomization to progression/recurrence or death. NADIM II PFS was retained only in endpoint-compatible sensitivity analysis and was not substituted for EFS in the primary pool. |
| OS | Time from randomization to death from any cause. |
| MPR | No more than 10% residual viable tumor in the resected primary tumor/tumor bed, with nodal assessment as specified by the trial. |
| pCR | No residual viable invasive tumor in the resected primary tumor and sampled regional lymph nodes (ypT0/Tis ypN0 or trial-equivalent). |
| Surgery | Receipt or protocol-defined attempt of curative-intent lung resection among all randomized participants. Completed resection and R0 resection were retained separately when definitions differed. |
| Safety | Grade ≥3 treatment-related adverse events and adverse-event-related treatment discontinuation using the trial-reported attribution, CTCAE version, population, and reporting window. |
| Postoperative initiation | Experimental-arm participants receiving at least one dose/cycle of the protocol-specified postoperative component divided by the stated randomized or modified-ITT experimental-arm denominator. This was descriptive and not a randomized estimate of the postoperative component’s independent effect. |

# 10. Risk of bias and statistical analysis

Risk of bias was assessed with the Cochrane RoB 2 framework across randomization, deviations from intended interventions, missing outcome data, outcome measurement, and selective reporting. Statistical methods, including restricted maximum-likelihood random-effects models, heterogeneity measures, modified Hartung–Knapp sensitivity intervals, absolute risk differences, NNT/NNH calculations, logit-transformed postoperative-initiation proportions, and exploratory trial-level correlations, are described in the main manuscript.

# 11. Audit limitations and verification status

The forensic reconstruction has three material limitations: (1) the exact historical database queries and source exports cannot be recovered from the retained files; (2) the historical duplicate-removal process cannot be independently reproduced; and (3) screening was reconstructed by one reviewer. Consequently, the 3,280 early exclusions are supported by aggregate counts rather than a retained row-by-row bibliographic ledger. The 60 report-level decisions are documented in the audit workbook.

Before final resubmission, the preferred verification step is an independent second-reviewer check of all 60 report-level decisions and, if access is available, de novo execution of the reconstructed Embase and CENTRAL strategies with saved exports and a reproducible duplicate log. Any resulting change should be propagated simultaneously to main-text Figure 1, the Methods, the Results, Supplementary Table S1, and the audit workbook.

# 12. Supplementary files cited by these methods

| **File** | **Contents** |
| --- | --- |
| Supplementary Table S1 | Endpoint-level report selection and extraction map for all 12 parent trials and seven endpoint domains, including report, cutoff, endpoint definition, population, and selection rationale. |
| Search and screening audit workbook | Search strategies; provenance; PRISMA count reconciliation; screening-code definitions; all 60 report-level decisions; included-report bibliography; update audit; corpus-accounting limitations; and formula-based QA checks. |
